# Predicting Depressive Symptoms Using GPS-Based Regional Data in Germany With the CORONA HEALTH App During the COVID-19 Pandemic: Cross-Sectional Study

**DOI:** 10.2196/53248

**Published:** 2024-12-03

**Authors:** Johanna-Sophie Edler, Michael Winter, Holger Steinmetz, Caroline Cohrdes, Harald Baumeister, Rüdiger Pryss

**Affiliations:** 1 Mental Health Research Unit Department of Epidemiology and Health Monitoring Robert Koch Institute Berlin Germany; 2 Department of Clinical Epidemiology and Biometry University of Würzburg Würzburg Germany; 3 Department of Business Administration Trier University Trier Germany; 4 Department of Clinical Psychology and Psychotherapy Ulm University Ulm Germany

**Keywords:** depression, COVID-19, mobile phone, geographic information systems, GPS-based data, mobile applications, mental health

## Abstract

**Background:**

Numerous studies have been conducted to predict depressive symptoms using passive smartphone data, mostly integrating the GPS signal as a measure of mobility. Environmental factors have been identified as correlated with depressive symptoms in specialized studies both before and during the pandemic.

**Objective:**

This study combined a data-based approach using passive smartphone data to predict self-reported depressive symptoms with a wide range of GPS-based environmental factors as predictors.

**Methods:**

The CORONA HEALTH app was developed for the purpose of data collection, and this app enabled the collection of both survey and passive data via smartphone. After obtaining informed consent, we gathered GPS signals at the time of study participation and evaluated depressive symptoms in 249 Android users with the Patient Health Questionnaire-9. The only GPS-based data collected were the participants’ location at the time of the questionnaire, which was used to assign participants to the nearest district for linking regional sociodemographic data. Data collection took place from July 2020 to February 2021, coinciding with the COVID-19 pandemic. Using GPS data, each dataset was linked to a wide variety of data on regional sociodemographic, geographic, and economic characteristics describing the respondent’s environment, which were derived from a publicly accessible database from official German statistical offices. Moreover, pandemic-specific predictors such as the current pandemic phase or the number of new regional infections were matched via GPS. For the prediction of individual depressive symptoms, we compared 3 models (ie, ridge, lasso, and elastic net regression) and evaluated the models using 10-fold cross-validation.

**Results:**

The final elastic net regression model showed the highest explained variance (*R*^2^=0.06) and reduced the dataset from 121 to 9 variables, the 3 main predictors being current COVID-19 infections in the respective district, the number of places in nursing homes, and the proportion of fathers receiving parental benefits. The number of places in nursing homes refers to the availability of care facilities for the elderly, which may indicate regional population characteristics that influence mental health. The proportion of fathers receiving parental benefits reflects family structure and work-life balance, which could impact stress and mental well-being during the pandemic.

**Conclusions:**

Passive data describing the environment contributed to the prediction of individual depressive symptoms and revealed regional risk and protective factors that may be of interest without their inclusion in routine assessments being costly.

## Introduction

### Background

Depression is a serious and widespread disease accompanied by a high individual burden [1]. Early detection of depressive symptoms facilitates care through early intervention and can thus prevent the development of severe symptoms such as suicidal ideation [2]. Therefore, knowledge of the current incidence and rapid recognition of trends in the prevalence of depressive symptoms is of great social relevance [3], for instance, for planning treatment strategies and identifying capacities.

To identify such trends in a timely manner, population-wide (broad) data collection in real time is pivotal. Collecting survey data requires the commitment of the participants, and they have to take the time and effort to answer questions. With the proliferation of smartphones, an alternative data source is available that does not require the effort of participants. Smartphone data have been used in research on mental health [4,5] and explicitly for depressive symptoms [6,7]. Analyses based on these smartphone data often aim to predict, for instance, depressive symptoms by including all available data [4,6,8,9]. A distinction is made between active and passive data collected via smartphones. Active smartphone data involve data that require the active involvement of the smartphone user (eg, survey data collected via the smartphone), and passive smartphone data include data that can be collected without the user’s involvement [4]. Passive smartphone data can be divided into activity data, social media data, and sensor data [10]. A whole body of evidence investigates the predictive power of activity data (eg, text messages sent, calls made, and screen activity), which are understood as objective measures of behavior [11]. It has been shown that some of these activity data are clearly related to depressive symptoms [12-15]. Social media data include information about posts on social networks, and these aspects of social interaction are also associated with depressive symptoms [16]. Smartphone sensor data involve the data measured with the sensors integrated in smartphones, such as GPS [10]. Studies often use GPS to analyze mobility behavior and have been able to identify correlations with depressive symptoms as well [4,7,10,14,17-20]. However, by including GPS sensor data in research on depressive symptoms, it is further possible to describe the participants’ environment in detail. Environmental factors include, for example, the sociodemographics of the population living in the surrounding area, economic conditions, social affairs, or living conditions (Table S1 in Multimedia Appendix 1). These environmental factors are external factors that affect people’s living conditions. For example, urbanization and population density are controversially discussed as risk factors for depressive symptoms [21]. In addition, different types of noise and air pollution were predominantly identified as positively related to depressive symptoms [21]. A poor home or building environment has been repeatedly identified as positively correlated with depressive symptoms, and the same applies to a lack of green areas [21].

This study was conducted during the COVID-19 pandemic. This was a time when people spent more time at home due to contact restrictions and lockdowns [22]. Researchers argue that environmental factors may have been particularly important for mental health during the pandemic, as more time was spent in those same environments [23-25]. This is also indicated by the results showing an association between housing conditions (eg, available space) and depressive symptoms during the COVID-19 pandemic [23,26]. For this reason, environmental factors should be included in studies on depressive symptoms (especially during the COVID-19 pandemic). In addition, integrating environmental factors allows for a better understanding of depressive symptoms, namely, to what extent environmental factors are risk and protective factors for depressive symptoms [27]. Furthermore, as some environmental factors such as sociodemographics (eg, employment rate and age distribution), economic conditions (eg, investments per employee), social affairs (eg, percentage of households with children), and living conditions (eg, percentage of 1-person households) can be passively measured via GPS data, the prediction and identification of depressive symptoms can be achieved with much lower costs, effort, and in a faster manner. However, it is unclear how accurately self-reported individual depressive symptoms can be predicted purely on the basis of GPS signals.

### Study Design

The aim of this study was to clarify to what extent environmental factors that can be passively measured via an individual’s location tracked by GPS explained the statistical variance in individual depressive symptoms. In contrast to many studies that have focused on single predictors only [13], this study used a comprehensive approach and integrated a wide range of data available through GPS smartphone data (eg, sociodemographics, economic conditions, social affairs, and living conditions). Since this study was conducted during the COVID-19 pandemic, GPS-based COVID-19–specific variables such as the pandemic phase and the regional COVID-19 infection count were integrated as well.

The analyses were based on a data-driven statistical approach that allowed us to analyze a large number of predictor variables for individual depressive symptoms in an exploratory fashion without overfitting the data. Hence, there was no a priori selection of included variables based on any assumed connection to depressive symptoms, but all available variables of the data source were included.

## Methods

### Sample and Procedure

Data collection took place by means of the developed CORONA HEALTH app, a cooperative project between the German universities of Würzburg, Ulm, and Regensburg, and the German Robert Koch Institute. The CORONA HEALTH app was specifically designed to explore factors during the COVID-19 pandemic and allows for both cross-sectional and longitudinal data analyses [28]; in this paper, we exclusively used cross-sectional data. Participants were recruited by means of public relation measures (eg, press releases and social networks). Participation in this study was voluntary, and participants did not receive incentives for participation. The inclusion criterion (for the analyzed study) was a minimum age of 18 years. In this study, only those data from smartphones with Android operating systems were analyzed. To participate, participants had to download the app and were then requested to provide informed consent (including use of GPS data). The study information provided was based on the quality standards set out by Beierle et al [29].

Based on the recorded GPS signal during survey participation, we assigned each participant to the nearest district, which was the smallest possible regional unit. The only GPS-based passive data collected were the participants’ location at the time of the questionnaire. This location was used to link participants to district-level sociodemographic and environmental data from official German statistical sources. For data protection reasons, the resolution of the location was reduced to an accuracy of 11.1 km. In Germany, the statistical offices of the counties and the federal government provide current average values for 160 indicators at the regional level in the so-called Regional Atlas [30]. All these available average values for population structure, housing conditions, and so forth, were assigned to each participant according to his or her respective district. Data were gathered from July 2020 to February 2021, coinciding with the COVID-19 pandemic. This period in Germany was marked by several waves of infections and accompanying contact-restricting measures and correspondingly restricted public life [22]. Based on the date of the first questionnaire completion, the COVID-19 incidence rate in the respective district and information on the pandemic phase [22] were determined and assigned to the individual dataset.

The conception and implementation of the app followed medical device regulations and were certified accordingly (see the study by Holfelder et al [31]). This study was approved by the ethics committee of the University of Würzburg (130/20-me).

Due to the inclusion of exclusively passive data, the dataset did not contain any individual sociodemographic information but rather the sociodemographic averages in the respective district. The following sociodemographic averages describe the sample: those aged 0-17 years accounted for 15% of the population (averaged across all districts), those aged 18-24 years accounted for 8.3%, those aged 25-44 years accounted for 29.7%, those aged 45-64 years accounted for 25.6%, and those older than 65 years accounted for 27.5%. It is important to note that while those aged 0-17 years were mentioned as comprising 15% of the population, this figure provides general context about the population, as individuals younger than 18 years were not included in the study sample. On average, 39.6% of the population in the districts graduated from school with a general matriculation standard, and an average of 6% did not have a school-leaving certificate.

The original sample had 1760 participants. Eighteen individuals were dropped in a plausibility check based on correspondence between similar items, straightlining, intraindividual response variability, and extreme outliers. Of the remaining sample of participants, 60.4% (1052/1741) had an Android operating system, and 28.1% (490/1741) consented to the collection of passive data. Of the remaining sample, only those districts with more than 20 participants were included to meet the condition of normally distributed data in the clusters. This resulted in a final sample of 249 participants. Details on the sample can be found in Table S1 in Multimedia Appendix 1.

### Measures

The primary outcome of this study was depressive symptoms, measured using the German version of the Patient Health Questionnaire-9 (PHQ-9) [32]. The PHQ-9 is a validated self-report instrument consisting of 9 items (eg, “Over the last week how often have you experienced little interest or pleasure in doing things?”), with responses on a 4-point scale (0=“Not at all” to 3=“Almost every day”). The total PHQ-9 score ranges from 0 to 27, with higher scores indicating more severe depressive symptoms. A score of 0-4 suggests minimal depressive symptoms; 5-9, mild symptoms; 10-14, moderate symptoms; 15-19, moderately severe symptoms; and 20-27, severe symptoms. The internal consistency of the PHQ-9 in this sample was excellent, with a Cronbach α=0.85.

The PHQ-9 is widely used in both clinical and general populations and was chosen as the outcome measure because it allows for the assessment of the severity of depressive symptoms in a continuous format, which aligns with the study’s objective to explore predictors of individual depressive symptoms. As a COVID-19 pandemic–specific variable, the number of infections per 100,000 inhabitants in the respective district at the time of assessment was included. These data were derived from official statistics reported by the Robert Koch Institute based on all laboratory-confirmed officially reported SARS-CoV-2 cases within the framework of the German Infection Protection Act [33]. In addition, the pandemic phase at the time of assessment was included in each case according to the classification by Schilling and colleagues [22] ranging from 1 (calendar weeks 10-20 in 2020) to 3 (calendar week 40 in 2020).

The respective regional environmental factors were determined using the district-level statistics of the Regional Atlas of the Federal Statistical Offices [30]. All variables available on the website on April 30, 2022, at the district level were included, with each construct included once. These included data on sociodemographics (eg, age group percentages in 2019, youth ratio in the regional population in 2021, percentage of the population without school-leaving qualifications in 2019, employment rate in 2020, disposable income per inhabitant in euros in 2020, and percentage of unemployed people in 2020), economy (eg, percentage of employed individuals by sector in 2019, investments per employee in thousand euros in 2019, and average length of stay of tourists in 2019), social affairs (percentage of households with children in 2011, percentage of children in care by age on March 1, 2021, proportion of fathers receiving parental benefits in 2014, and places in nursing homes per 1000 inhabitants aged 65 years and older in 2020), and living environment (eg, percentage of 1-person households in 2011, population density of inhabitants per square kilometer in 2020, percentage of area by use in 2015, and consumption-based charge for drinking water supply per cubic meter in 2019). A complete list of variables is shown in Table S1 in Multimedia Appendix 2. Due to the district clusters, the respective districts were included in the analyses as dummy variables. In addition, the pandemic phases were included as dummy variables. In total, the dataset included 121 variables.

### Data Analyses

Using GPS coordinates, each participant was assigned to a district. To control for the nested data, the respective districts were included in the analysis as dummy variables. Two participants who had a GPS location outside Germany were excluded. The data contained only 3 missing values (in the depressive symptoms variable); these were replaced using a single imputation with 500 iterations using classification and regression trees (cart method) with the R package “mice” (ie, the mice package for multivariate imputation by chained equations was developed by Stef van Buuren and is supported by Utrecht University) [34]. Metric variables were *z* standardized.

**Figure 1:**
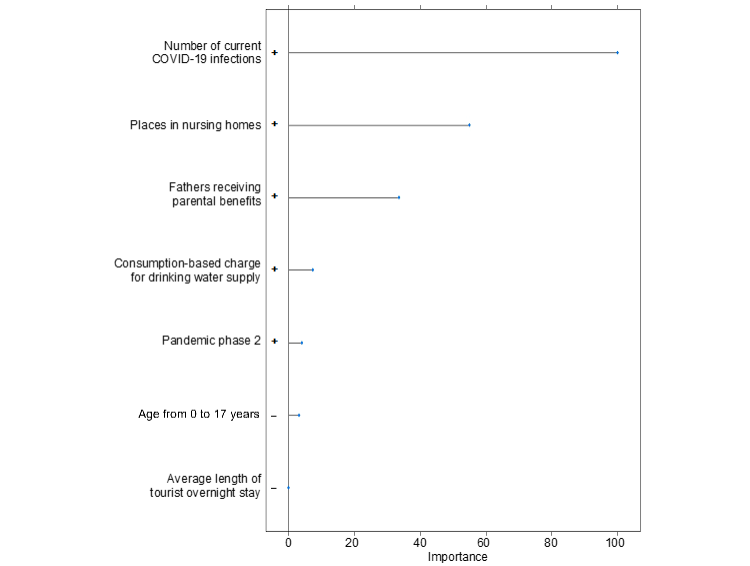
Total variable importance as an indicator of the contribution to reduce the estimation error in the prediction of depressive symptoms in the lasso regression model.

**Figure 2:**
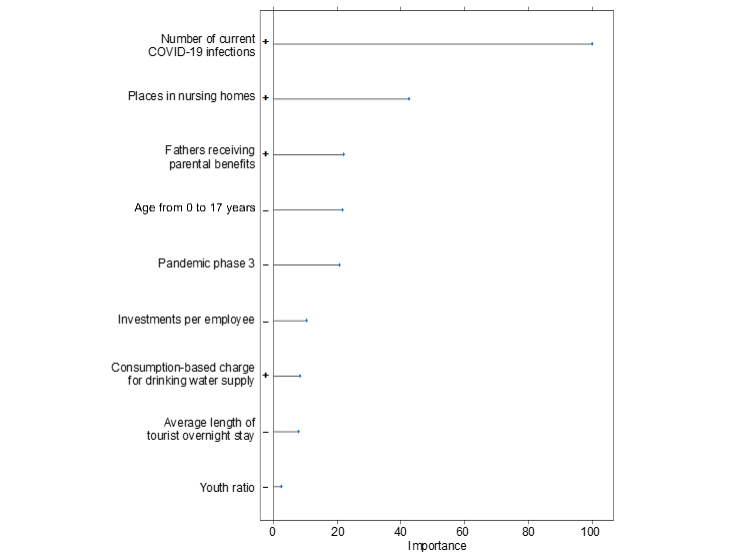
Total variable importance as an indicator of the contribution to reduce the estimation error in the prediction of depressive symptoms in the elastic net regression model.

For the prediction of individual depressive symptoms, we chose to compare ridge, lasso, and elastic net regression models [35]. The predictive performance, signaling the best fit, relied on the root-mean-square error, mean absolute error, and explained variance (*R*^2^). Using a grid search, we implemented hyperparameter optimization. The analyses included automated variable selection. This allowed for a statistical check of which variables remained in the model with the best predictive performance, that is, the lowest degree of prediction errors [36]. To avoid overfitting, we split the dataset into a training (70%) and a test dataset (30%). In the training dataset, the final model was calculated using 10-fold cross-validation with 5-fold repetition [35]. In the test dataset, the model was evaluated for its predictive performance in a sample that had not been part of the model training. The result was a ranking by variable importance (Figures 1 and 2). Variables were ranked based on their contribution to the prediction of individual depressive symptoms.

### Ethical Considerations

The conception and implementation of the app followed medical device regulations and were certified accordingly [31]. The original data collection for this study was approved by the ethics committee of the University of Würzburg (130/20-me). This approval explicitly covered both initial data collection and any potential future secondary analyses using the collected data. As such, no additional institutional review board approval or exemption was necessary for the present secondary analysis. In accordance with local regulations and institutional guidelines, the secondary use of existing, anonymized data does not require a separate review or approval. Specifically, the main institutional review board votum issued during the original data collection already included provisions for future secondary analyses. This is in line with German medical research standards, which state that when data are collected with informed consent that includes provisions for future use, and the data are anonymized, additional ethical approval is not required for secondary analyses (see Article 89 of the German Data Protection Regulation). All procedures adhered to institutional ethical standards for research involving human subjects, and all participant data were fully anonymized to protect privacy. Participation was voluntary, and no financial compensation was provided.

## Results

The comparison of the individual models (ridge, lasso, and elastic net regression) showed the following differences: in the prediction of individual depressive symptoms, lasso regression had the smallest average root-mean-square error (5.59). The explained variance was second to highest (*R*^2^=0.05). The elastic net regression produced the highest explained variance (*R*^2^=0.06; Table 1). The listed characteristic values showed lasso and elastic net regression as the analyses with the best fit. Lasso regression reduced the dataset from 121 variables to 7 variables, and elastic net regression reduced it from 121 to 9 variables.

The lasso and elastic net regression models shared the first 3 predictors. The most important predictor was the number of current COVID-19 infections in the respective district. The second was the number of places in nursing homes, and the third was the proportion of fathers receiving parental benefits. Both models also included the 3 variables population share of the 0-17 years age group, average length of stay of tourists, and the consumption-based charge for drinking water.

Lasso regression showed the second pandemic phase as a relevant predictor (Figure 1). Elastic net regression further listed (in descending variable importance) the third pandemic phase, investment per employee and the youth ratio as relevant predictors (Figure 2).

**Table 1:** Model fit indices for predicting depressive symptoms resulting from stepwise, ridge, lasso, and elastic net regression based on 50 replicate samples^a^.

			Minimum		1st quantile		Median		Mean		3rd quantile		Maximum
**MAE^b^**													
	Ridge	3.628		4.127		4.515		4.448		4.746		5.524	
	Lasso	3.606		4.231		4.440		4.464		4.643		5.717	
	Elastic net	3.605		4.162		4.506		4.483		4.741		5.764	
**RMSE^c^**													
	Ridge	4.165		5.071		5.626		5.594		6.080		7.373	
	Lasso	4.216		5.244		5.630		5.585		5.942		7.104	
	Elastic net	4.169		5.219		5.677		5.618		5.912		7.162	
***R^2^***													
	Ridge	0.000		0.003		0.019		0.048		0.079		0.228	
	Lasso	0.000		0.003		0.019		0.053		0.052		0.366	
	Elastic net	0.000		0.009		0.034		0.063		0.083		0.415	

^a^Overall, elastic net regression had the best model fit due to the largest variance explained (*R²*), and lasso regression had the best model fit when including the smallest mean absolute error and root-mean-square error.

^b^MAE: mean absolute error.

^c^RMSE: root-mean-square error.

## Discussion

### Principal Findings

This study aimed to identify correlates of depressive symptoms based on regional factors allocated via GPS (passive data). It should be noted that the only GPS-based passive data collected were the participants’ location at the time of the questionnaire, which was then used to assign participants to regional sociodemographic and environmental data based on their district. For this purpose, we used a passive data collection method. This had the advantage of integrating a wide range of data without any effort on the part of the participants.

The results helped identify regional and freely available variables as risk and protective factors for individual depressive symptoms. The identified risk and protective factors are presented as follows, starting with the 3 aspects that had the greatest relevance in both models.

The weekly number of cases of COVID-19 infections in the respective district was positively associated with higher individual depressive symptoms. In many cases, high COVID-19 case numbers were associated with contact restrictions in everyday life imposed by law or by the population itself [37]. A connection between these contact restrictions and individual depressive symptoms has been discussed repeatedly [38-41]. Different arguments were used to explain this connection. The reduction in social contact promoted loneliness, which has been identified as a risk factor for individual depressive symptoms [42,43]. In addition, an increase in misconduct and home violence has been reported [44]. Moreover, the high number of cases might have been associated with increased fear of infection, which increased substantially during the pandemic [41], a lack of care capacities, or a severe course of the disease [45]. This could be related to individual depressive symptoms as an additional burden [46].

The proportion of fathers receiving a parental allowance was a risk factor for higher individual depressive symptoms. Parental allowance is a financial state benefit to mothers or fathers in Germany that is granted for a certain period during which the parent is not or only partially employed and devotes himself or herself to the care of their child. For methodological reasons, it was not possible to derive any causal associations from the results of our analysis. However, one possible explanation could be that paternal receipt of parental allowance might be an indicator for those families in which both parents are employed. These families were thus dependent on the functioning of the childcare infrastructure and therefore particularly challenged by the double burden of working from home and homeschooling for the duration of the pandemic. In a different study, increased individual depressive symptoms were also observed in parents of underage children during the COVID-19 pandemic, which also indicates the special burden on families [47].

In addition, the pandemic phase was a relevant factor for individual depressive symptoms. Phase 2 of the pandemic was positively related to individual depressive symptoms. The second phase of the pandemic, from the end of May to the end of September 2020, was accompanied by the opening of restaurants, gradual school openings, and the lifting of travel restrictions [22]. It is possible that with this return to regular daily life, depressive symptoms became more noticeable. In other words, it could be that it was only at the moment when a person was no longer able to cope with demands that he or she became aware of his or her own mental health condition. This could have been the case for those affected by depressive symptoms after the contact restrictions were removed and they returned to everyday demands. The same reasoning could apply to the negative correlation between phase 3 of the pandemic and individual depressive symptoms. The third pandemic phase was characterized by a nationwide partial lockdown at the beginning of November 2021 with tightened contact restrictions and a nationwide lockdown with moderately strict regulations from mid-December and the Christmas holidays [22]. Due to contact restrictions and holidays, there were fewer day-to-day demands to attend to during the period, such as homeschooling, daily logistics due to travel, and social obligations. In the case of individual depressive symptoms associated with reduced drive and anhedonia [48], these individual depressive symptoms would have been less noticeable during the lockdown, as there were fewer events to manage anyway.

In contrast to previous findings, a high proportion of the 0-17 years age group and a high youth ratio were associated with lower levels of individual depressive symptoms. In contrast, numerous studies have shown that stress and individual depressive symptoms in families increased during the pandemic [47,49,50]. Since only proportions of the population and not the absolute numbers were collected, a large share of the 0-17 years age group can also mean that the proportion of families was correspondingly large and thus the proportion of people living alone correspondingly smaller. Research showed that 1-person households correlated with lower well-being compared with households with 2-4 people during the pandemic [51]. Furthermore, correlations between loneliness and individual depressive symptoms were also found [52].

In contrast to the present results, previous studies showed a correlation between nearby green spaces [25] or the quality of housing conditions (>60 m^2^, view from the windows, and poor indoor quality) [23] and lower depressive mood. A systematic review confirmed this and identified poor housing, lack of green spaces, and both noise and air pollution as environmental factors that were associated with depressive mood across studies [21]. The reason for the deviation of the present results could be that availability alone was not the decisive predictor for the association with depressive symptoms. Using the example of green spaces, research conducted during the COVID-19 pandemic, for example, showed that although natural areas were visited more often, visits to parks decreased [25]. The authors further discussed to what extent it was the time spent in green spaces that was related to low depressive symptoms or whether the green space was explicitly visited to participate in sports or to maintain social contacts and whether these were actually relevant predictors [25]. Sonnentag et al [53] found that the psychological mechanisms underlying recovery seem to be enhanced in natural environments.

### Strengths and Limitations

A central strength of this study is the application of a data-driven statistical approach that allowed us to analyze a large number of predictor variables in an exploratory fashion without overfitting the data. By these means, the approach allowed us to investigate which environmental factors had predictive power. The collection of passive data via smartphone made it possible to reach participants despite pandemic-related contact restrictions and to make a corresponding statement about the included predictors for the COVID-19 pandemic period.

The cross-sectional study design of this study was limiting; statements about causal relationships were therefore not possible. In addition, the final dataset was very small, which might have weakened the significance of the analyses. A limitation that other studies have also observed [12] is the limited predictive power of individual depressive symptoms based on the passive data used. The use of GPS data was also a limitation, as it included only the location at the time of questionnaire processing. This was not the same as the place of residence of the respective person. How long the respective person was exposed to the general conditions could therefore not be inferred from the data. In addition, the informative value of the GPS data regarding individual environmental conditions was limited due to the regional resolution: since large variances in the average values were possible, it was of course not possible to draw conclusions about individual cases. Moreover, the image of the environment was only very roughly described. However, it represented the smallest possible regional size at which data from statistical offices could be collected nationwide. In addition, a higher spatial resolution, for example, per block of houses, would make it impossible to anonymize the data, as too few people would remain in the respective cluster. The validity of this study is further limited by the nonrepresentative dataset. As this analysis was limited to data from Android smartphones and required agreement to the collection of passive data, selection bias could not be ruled out. This must be taken into account when evaluating the results of this study. In addition, neither changes in predictors over time nor long-term data collection was considered to depict cause and effect more validly. This study can therefore make statements only about correlations and not about cause and effect.

### Conclusions

This study showed that regional average data on socioeconomics and living environment seemed to be limited predictors of individual depressive symptoms; for the pandemic period, individual depressive symptoms were predicted, although the predictors had a rather low explanatory value and are not transferable to postpandemic periods. This suggests that the spatial resolution would need to be modified or that the dataset would need to be supplemented with additional data sources. Nevertheless, the results help classify freely available average data as risk or protective factors for individual depressive symptoms.

## Appendix

Multimedia Appendix 1 Descriptive statistics of the present sample of 249 German-speaking adults across districts.

Multimedia Appendix 2 A list of variables from the Regional Atlas of Germany detailing sociodemographic, economic, and environmental data used in the study.

## Abbreviations

PHQ-9: Patient Health Questionnaire-9

## References

[1] Lépine JP, Briley M (2011). The increasing burden of depression. Neuropsychiatr Dis Treat.

[2] Wahlbeck K (2015). Public mental health: the time is ripe for translation of evidence into practice. World Psychiatry.

[3] Fried EI, Nesse RM (2015). Depression sum-scores don't add up: why analyzing specific depression symptoms is essential. BMC Med.

[4] Onnela JP, Rauch SL (2016). Harnessing smartphone-based digital phenotyping to enhance behavioral and mental health. Neuropsychopharmacology.

[5] Torous J, Onnela JP, Keshavan M (2017). New dimensions and new tools to realize the potential of RDoC: digital phenotyping via smartphones and connected devices. Transl Psychiatry.

[6] He X (2022). Depression diagnosis and forecast based on mobile phone sensor data.

[7] Seppälä J, De Vita I, Jämsä T, Miettunen J, Isohanni M, Rubinstein K, Feldman Y, Grasa E, Corripio I, Berdun J, D'Amico E, Bulgheroni M, M-RESIST Group (2019). Mobile phone and wearable sensor-based mHealth approaches for psychiatric disorders and symptoms: systematic review. JMIR Ment Health.

[8] Opoku Asare K, Terhorst Y, Vega J, Peltonen E, Lagerspetz E, Ferreira D (2021). Predicting depression from smartphone behavioral markers using machine learning methods, hyperparameter optimization, and feature importance analysis: exploratory study. JMIR Mhealth Uhealth.

[9] Shakeri Hossein Abad Z, Kline A, Sultana M, Noaeen M, Nurmambetova E, Lucini F, Al-Jefri M, Lee J (2021). Digital public health surveillance: a systematic scoping review. NPJ Digit Med.

[10] Birk RH, Samuel G (2022). Digital phenotyping for mental health: reviewing the challenges of using data to monitor and predict mental health problems. Curr Psychiatry Rep.

[11] Place S, Blanch-Hartigan D, Rubin C, Gorrostieta C, Mead C, Kane J, Marx BP, Feast J, Deckersbach T, Pentland AS, Nierenberg A, Azarbayejani A (2017). Behavioral indicators on a mobile sensing platform predict clinically validated psychiatric symptoms of mood and anxiety disorders. J Med Internet Res.

[12] Currey D, Torous J (2022). Digital phenotyping correlations in larger mental health samples: analysis and replication. BJPsych Open.

[13] Dogan E, Sander C, Wagner X, Hegerl U, Kohls E (2017). Smartphone-based monitoring of objective and subjective data in affective disorders: where are we and where are we going? Systematic review. J Med Internet Res.

[14] Opoku Asare K, Moshe I, Terhorst Y, Vega J, Hosio S, Baumeister H, Pulkki-Råback L, Ferreira D (2022). Mood ratings and digital biomarkers from smartphone and wearable data differentiates and predicts depression status: a longitudinal data analysis. Pervasive Mobile Comput.

[15] Wang R, Wang W, da Silva A, Huckins JF, Kelley WM, Heatherton TF, Campbell AT (2018). Tracking depression dynamics in college students using mobile phone and wearable sensing. Proc ACM Interactive Mobile Wearable Ubiquitous Technol.

[16] Liu D, Feng XL, Ahmed F, Shahid M, Guo J (2022). Detecting and measuring depression on social media using a machine learning approach: systematic review. JMIR Ment Health.

[17] Moshe I, Terhorst Y, Opoku Asare K, Sander LB, Ferreira D, Baumeister H, Mohr DC, Pulkki-Råback L (2021). Predicting symptoms of depression and anxiety using smartphone and wearable data. Front Psychiatry.

[18] Rohani DA, Faurholt-Jepsen M, Kessing LV, Bardram JE (2018). Correlations between objective behavioral features collected from mobile and wearable devices and depressive mood symptoms in patients with affective disorders: systematic review. JMIR Mhealth Uhealth.

[19] Saeb S, Zhang M, Kwasny MM, Karr CJ, Kording K, Mohr DC (2015). The relationship between clinical, momentary, and sensor-based assessment of depression. Int Conf Pervasive Comput Technol Healthc.

[20] Sükei E, Norbury A, Perez-Rodriguez MM, Olmos PM, Artés A (2021). Predicting emotional states using behavioral markers derived from passively sensed data: data-driven machine learning approach. JMIR Mhealth Uhealth.

[21] Rautio N, Filatova S, Lehtiniemi H, Miettunen J (2018). Living environment and its relationship to depressive mood: a systematic review. Int J Soc Psychiatry.

[22] Schilling J, Buda S, Fischer M (2021). Retrospektive Phaseneinteilung der COVID-19-Pandemie in Deutschland bis Februar.

[23] Amerio A, Brambilla A, Morganti A, Aguglia A, Bianchi D, Santi F, Costantini L, Odone A, Costanza A, Signorelli C, Serafini G, Amore M, Capolongo S (2020). COVID-19 lockdown: housing built environment's effects on mental health. Int J Environ Res Public Health.

[24] Groot J, Keller A, Joensen A, Nguyen TL, Nybo Andersen AM, Strandberg-Larsen K (2022). Impact of housing conditions on changes in youth's mental health following the initial national COVID-19 lockdown: a cohort study. Sci Rep.

[25] Lõhmus M, Stenfors CUD, Lind T, Lauber A, Georgelis A (2021). Mental health, greenness, and nature related behaviors in the adult population of stockholm county during COVID-19-related restrictions. Int J Environ Res Public Health.

[26] Ramiz L, Contrand B, Rojas Castro MY, Dupuy M, Lu L, Sztal-Kutas C, Lagarde E (2021). A longitudinal study of mental health before and during COVID-19 lockdown in the French population. Global Health.

[27] Han J, Zhang Z, Mascolo C, Andre E, Tao J, Zhao Z, Schuller BW (2021). Deep learning for mobile mental health: challenges and recent advances. IEEE Signal Process Mag.

[28] Beierle F, Schobel J, Vogel C, Allgaier J, Mulansky L, Haug F, Haug J, Schlee W, Holfelder M, Stach M, Schickler M, Baumeister H, Cohrdes C, Deckert J, Deserno L, Edler J, Eichner FA, Greger H, Hein G, Heuschmann P, John D, Kestler HA, Krefting D, Langguth B, Meybohm P, Probst T, Reichert M, Romanos M, Störk S, Terhorst Y, Weiß M, Pryss R (2021). Corona health-a study- and sensor-based mobile app platform exploring aspects of the COVID-19 pandemic. Int J Environ Res Public Health.

[29] Beierle F, Tran VT, Allemand M, Neff P, Schlee W, Probst T, Pryss R, Zimmermann J (2018). Context data categories and privacy model for mobile data collection apps. Procedia Comput Sci.

[30] Statistical Offices of the Federal and State Governments Joint statistics portal.

[31] Holfelder M, Mulansky L, Schlee W, Baumeister H, Schobel J, Greger H, Hoff A, Pryss R (2021). Medical device regulation efforts for mHealth apps during the COVID-19 pandemic—an experience report of corona check and corona health. J Multidisciplinary Sci J.

[32] Löwe B (2002). PHQ-D manual. Komplettversion und Kurzform.

[33] Federal Ministry of Justice Act on the prevention and control of infectious diseases in humans.

[34] van Buuren S Multivariate imputation by chained equations.

[35] James G (2013). An Introduction to Statistical Learning With Applications in R.

[36] Zou H, Hastie T (2005). Regularization and variable selection via the elastic net. J Royal Statistical Soc B.

[37] Borkowski P, Jażdżewska-Gutta M, Szmelter-Jarosz A (2021). Lockdowned: everyday mobility changes in response to COVID-19. J Transp Geogr.

[38] Benke C, Autenrieth LK, Asselmann E, Pané-Farré CA (2020). Lockdown, quarantine measures, and social distancing: associations with depression, anxiety and distress at the beginning of the COVID-19 pandemic among adults from Germany. Psychiatry Res.

[39] Bonati M, Campi R, Segre G (2022). Psychological impact of the quarantine during the COVID-19 pandemic on the general European adult population: a systematic review of the evidence. Epidemiol Psychiatr Sci.

[40] Mata J, Wenz A, Rettig T, Reifenscheid M, Möhring K, Krieger U, Friedel S, Fikel M, Cornesse C, Blom AG, Naumann Elias (2021). Health behaviors and mental health during the COVID-19 pandemic: a longitudinal population-based survey in Germany. Soc Sci Med.

[41] Petzold MB, Bendau A, Plag J, Pyrkosch L, Mascarell Maricic L, Betzler F, Rogoll J, Große J, Ströhle A (2020). Risk, resilience, psychological distress, and anxiety at the beginning of the COVID-19 pandemic in Germany. Brain Behav.

[42] Hoffart A, Johnson SU, Ebrahimi OV (2020). Loneliness and social distancing during the COVID-19 pandemic: risk factors and associations with psychopathology. Front Psychiatry.

[43] Palgi Y, Shrira A, Ring L, Bodner E, Avidor S, Bergman Y, Cohen-Fridel S, Keisari S, Hoffman Y (2020). The loneliness pandemic: loneliness and other concomitants of depression, anxiety and their comorbidity during the COVID-19 outbreak. J Affect Disord.

[44] Thiel F, Büechl VCS, Rehberg F, Mojahed A, Daniels JK, Schellong J, Garthus-Niegel S (2022). Changes in prevalence and severity of domestic violence during the COVID-19 pandemic: a systematic review. Front Psychiatry.

[45] Schmitz A, Garten C, Kühne S, Brandt M (2022). Worries about inadequate medical treatment in case of a COVID-19 infection: the role of social inequalities, COVID-19 prevalence and healthcare infrastructure. BMC Public Health.

[46] Ingram RE, Luxton DD, Hankin BL, Abela JRZ (2005). Vulnerability-stress models. Development of Psychopathology: A Vulnerability-Stress Perspective.

[47] Johnson M, Skjerdingstad Nora, Ebrahimi OV, Hoffart U, Johnson SU (2022). Parenting in a pandemic: parental stress, anxiety and depression among parents during the government-initiated physical distancing measures following the first wave of COVID-19. Stress Health.

[48] World Health Organization (2019). Pocket Guide to the ICD-10 Classification of Mental Disorders.

[49] Calvano C, Engelke L, Di Bella J, Kindermann J, Renneberg B, Winter SM (2022). Families in the COVID-19 pandemic: parental stress, parent mental health and the occurrence of adverse childhood experiences-results of a representative survey in Germany. Eur Child Adolesc Psychiatry.

[50] Adams EL, Smith D, Caccavale LJ, Bean MK (2021). Parents are stressed! Patterns of parent stress across COVID-19. Front Psychiatry.

[51] Chen D, Wang Y (2021). Inequality-related health and social factors and their impact on well-being during the COVID-19 pandemic: findings from a national survey in the UK. Int J Environ Res Public Health.

[52] Werner AM, Tibubos AN, Mülder LM, Reichel JL, Schäfer M, Heller S, Pfirrmann D, Edelmann D, Dietz P, Rigotti T, Beutel ME (2021). The impact of lockdown stress and loneliness during the COVID-19 pandemic on mental health among university students in Germany. Sci Rep.

[53] Sonnentag S, Venz L, Casper A (2017). Advances in recovery research: what have we learned? What should be done next?. J Occup Health Psychol.

