# Supplementary material for: Predicting Depressive Symptoms Using GPS-Based Regional Data in Germany With the CORONA HEALTH App During the COVID-19 Pandemic: Cross-Sectional Study
Source: Interact J Med Res. 2024 Dec 3;13:e53248. doi: 10.2196/53248 (PMC11653045; doi:10.2196/53248)
Supplement: Multimedia Appendix 1 [file ijmr_v13i1e53248_app1.docx]

Table S1. Descriptive statistics of the present sample of N=249 German speaking adults in total across districts.

| Variables | *M* | *(SD)* | *Median* | *Min.* | *Max.* |
| --- | --- | --- | --- | --- | --- |
| PHQ-9 | 7.40 | 5.60 | 6 | 0 | 26 |
| COVID-19 pandemic | | | | | |
| New infections | 57.97 | 67.10 | 15 | 2 | 233 |
| Sociodemographics | | | | | |
| Age groups in 2019^a^ |  |  |  |  |  |
| 0-17 years | 15.01 | 1.75 | 16 | 12 | 17 |
| 18-24 years | 8.33 | 2.08 | 7 | 7 | 12 |
| 25-44 years | 29.70 | 3.30 | 31 | 23 | 33 |
| 45-64 years | 25.57 | 2.32 | 25 | 23 | 30 |
| from 65 years | 19.19 | 1.29 | 19 | 17 | 21 |
| Youth ratio in 2021^b^ | 27.52 | 3.36 | 29 | 22 | 32 |
| General higher education entrance qualification in 2021^a^ | 39.58 | 10.11 | 40 | 10 | 53 |
| Without school leaving qualification in 2019^a^ | 6.02 | 1.43 | 6 | 4 | 8 |
| Employment rate in 2020^a^ | 59.84 | 3.22 | 59 | 56 | 65 |
| Disposable income per inhabitant in 2020^b^ | 25138.04 | 3488.70 | 25808 | 21327 | 32039 |
| Proportion of unemployment benefit II recipients in 2019^a^ |  |  |  |  |  |
| up to 24 years | 8.79 | 5.40 | 8 | 2 | 16 |
| from 55 years | 6.90 | 3.29 | 5 | 1 | 11 |
| Economy | | | | | |
| Share of employed persons by sector in 2019^a^ |  |  |  |  |  |
| public and other services, education and health care | 36.15 | 7.88 | 38 | 24 | 48 |
| finance, insurance, trade, real estate and housing | 21.37 | 4.74 | 24 | 15 | 28 |
| trade, transport, hospitality, information and communication | 27.35 | 2.61 | 26 | 25 | 32 |
| service sectors | 85.87 | 5.83 | 89 | 70 | 90 |
| manufacturing industry | 7.80 | 3.36 | 6 | 5 | 17 |
| producing industry | 13.09 | 4.89 | 11 | 9 | 26 |
| construction industry | 3.21 | 1.75 | 3 | 1 | 7 |
| agriculture, forestry and fisheries | 0.24 | 0.82 | 0 | 0 | 3 |
| Investments per employee in 2019^c^ | 13.76 | 7.41 | 10 | 6 | 29 |
| Average length of tourist stay in 2019^d^ | 1.68 | 0.47 | 2 | 1 | 2 |
| Social affairs | | | | | |
| Households with children in 2011^a^ | 25.21 | 5.85 | 24 | 20 | 40 |
| Childcare rate 0 to 2 years on 01.03.2021^a^ | 39.60 | 5.42 | 44 | 29 | 46 |
| Childcare rate 3 to 5 years on 01.03.2021^a^ | 91.79 | 2.05 | 92 | 89 | 95 |
| Fathers receiving parental benefits in 2014^a^ | 40.17 | 6.95 | 37 | 26 | 52 |
| Places in nursing homes in 2020^e^ | 53.08 | 17.85 | 48 | 31 | 82 |
| Living environment | | | | | |
| One-person household in 2011^a^ | 45.87 | 7.41 | 49 | 29 | 51 |
| Population density in 2020^f^ | 2699.96 | 1607.34 | 2446 | 167 | 4777 |
| Share of area in 2015^a^ |  |  |  |  |  |
| agriculture | 22.28 | 18.05 | 22 | 4 | 61 |
| settlement | 42.73 | 15.26 | 46 | 7 | 58 |
| recreation | 8.61 | 4.34 | 8 | 0 | 13 |
| traffic | 12.61 | 3.06 | 12 | 6 | 16 |
| forest | 12.80 | 5.61 | 15 | 4 | 21 |
| Consumption-based charge for drinking water in 2019^g^ | 1.32 | 0.47 | 1 | 1 | 2 |
| Note: ^a^ in percent;  ^b^ per year in Euro;  ^c^ in thousand Euro;  ^d^ in days;  ^e^ per 1,000 inhabitants aged 65 and over;  ^f^ inhabitants per sqkm;  ^g^ in Euro per m^3.^ | | | | | |
