# Supplementary material for: Predicting Depressive Symptoms Using GPS-Based Regional Data in Germany With the CORONA HEALTH App During the COVID-19 Pandemic: Cross-Sectional Study
Source: Interact J Med Res. 2024 Dec 3;13:e53248. doi: 10.2196/53248 (PMC11653045; doi:10.2196/53248)
Supplement: Multimedia Appendix 2 [file ijmr_v13i1e53248_app2.docx]

Table S1. Variable list of the Regional Atlas of Germany produced by the Federal Statistical Office and the federal states.

| **Sociodemographics**   - population share of 0-17-year-olds in 2019 - population share of 18-24-year-olds in 2019 - population share of 25-44-year-olds in 2019 - population share of 45-64-year-olds in 2019 - population share of those aged 65 or older in 2019 - youth ratio in 2021 - old-age ratio in 2019 - average age of mother at birth of 1st child in 2019 - percentage of the population with general higher education entrance qualification in 2021 - percentage of the population without school leaving qualification in 2019 - employment rate in 2020 - share of 15-24-year-olds in the unemployed population in 2021 - share of 55-64-year-olds in the unemployed population in 2021 - proportion of children receiving financial social assistance in 2019 - share of those 55 years old or older in unemployment benefit II recipients in 2019 - proportion of those 24 years old or younger in unemployment benefit II recipients in 2019 - share of long-term unemployed in 2021 - share of foreigners in total population in 2019 - population share of 0-19-year-olds with a migration background in 2011 - population share of 20-59-year-olds with a migration background in 2011 - population share of those 60 years old or older with a migrant background in 2011 - naturalisation rate in 2019 - share of foreigners in the unemployed in 2021 - disposable income per inhabitant in euros in 2020 - basic income support rate for women aged 65 and over in 2021 - basic income rate of men aged 65 or older in 2021 - basic income support rate due to reduced earning capacity in 2019 - rate of employable SGB II beneficiaries for men in 2019 - rate of employable SGB II beneficiaries for women in 2019 - percentage of unemployed people in 2020 | **Politics**   - voter turnout in the Bundestag election in 2017 - turnout in the European Election in 2019 - vote share in the AfD European Election in 2019 - vote share in the CDU CSU European Election in 2019 - vote share in the DIE LINKE European Election in 2019 - vote share in the FDP European Election in 2019 - vote share in the GRÜNE European Election in 2019 - vote share in the SPD European Election in 2019 - AfD second vote share in the Bundestag election in 2017 - CDU CSU second vote share in the Bundestag election in 2017 - DIE LINKE second vote share in the Bundestag election in 2017 - FDP second vote share in the Bundestag election in 2017 - GRÜNE second vote share in the Bundestag election in 2017 - SPD second vote share in the Bundestag election in 2017   **Social affairs**   - percentage of households with children in 2011 - 0-2-year-old children in daycare facilities in 2021 - 3-5-year-old children in daycare facilities in 2021 - childcare rate for 0-2-year-olds in 2021 - childcare rate for 3-5-year-olds in 2021 - proportion of fathers receiving parental benefits in 2014 - hospital bed density in 2021 - places in nursing homes per 1,000 inhabitants for those aged 65 or over in 2020 - persons in need of care per 1,000 inhabitants for those aged 65 or over in 2021 - staff per 100 persons in need of outpatient care in 2021 - staff per 100 persons in need of full inpatient care in 2021 - proportion of men among pedagogical staff in child daycare facilities in 2021 |
| --- | --- |
| **Economy**   - employment density in 2019 - minimum protection rate in 2019 - share of employed persons in the construction industry in 2019 - share of employed persons in service sectors in 2019 - share of employed persons in the finance, insurance, real estate, and housing industries in 2019 - share of employed persons in the trade, transport, hotels and restaurants, information and communication industries in 2019 - share of employed persons in the agriculture, forestry and fishing industries in 2019 - proportion of employed persons in the public service and other services, education, and health industries in 2019 - share of employed persons in the producing sector in 2019 - share of employed persons in the manufacturing sector in 2019 - federal, state and local government employees per 1,000 inhabitants in 2019 - investments per employee in thousand euros in 2019 - gross domestic product per hour worked in 2017 - gross domestic product per person employed in 2019 - gross domestic product per inhabitant in 2020 - change in gross domestic product compared to that of the previous year in 2019 - gross wages per employee in 2019 - gross value added in the construction industry in 2019 - gross value added in service sectors in 2019 - gross value added in the finance, insurance, real estate, and housing industries in 2019 - gross value added in the trade, transport, hotels and restaurants, information and communication industries in 2019 - gross value added in the agriculture, forestry, and fishery industries in 2019 - gross value added in the public services and other services, education, and health sectors in 2019 - gross value added in the producing sector in 2019 - corporate insolvencies per 10,000 taxable companies in 2019 - average length of stay of tourists in 2019 - total amount of income per taxpayer in 2017 - business registrations per 10,000 inhabitants in 2020 | **Living environment**   - percentage of one-person households in 2011 - proportion of new residential buildings with 1 or 2 dwellings in 2019 - population density of inhabitants per square kilometre in 2020 - population development per year per 10,000 inhabitants in 2019 - average household size in 2021 - net migration per 10,000 inhabitants in 2019 - average farm size in 2016 - water delivery per inhabitant per day (in litres) in 2016 - consumption-based charge for drinking water supply per m^3^ in 2019 - consumption-independent charge for drinking water supply per m3 in 2019 - share of land for agriculture in 2015 - share of land for settlement in 2015 - share of land for sport, leisure and recreation in 2015 - share of area for transport in 2015 - share of area for forest in 2015 - share of organic farming in 2020 - cattle per 100 ha of utilised agricultural area in 2016 - pigs per 100 ha of utilised agricultural area in 2016 - car ownership per 1000 inhabitants in 2021 - number of those injured in road traffic accidents per 100,000 inhabitants in 2019 - number of fatalities in road accidents per 100 000 inhabitants in 2019 - number of road traffic accidents per 10 000 inhabitants in 2019 - number of road traffic accidents per 10 000 motor vehicles in 2019 - number of overnight stays per inhabitant in 2019 - amount of household waste per inhabitant in 2019 |
| *Note:* All county-level variables available on the Federal Statistical Office's website in the Regional Atlas on 30 April 2022 were considered, with each construct considered once. | |
